# Assessment of the Possible Association of Air Pollutants PM_10_, O_3_, NO_2_ With an Increase in Cardiovascular, Respiratory, and Diabetes Mortality in Panama City: A 2003 to 2013 Data Analysis

**DOI:** 10.1097/MD.0000000000002464

**Published:** 2016-01-15

**Authors:** Julio Zúñiga, Musharaf Tarajia, Víctor Herrera, Wilfredo Urriola, Beatriz Gómez, Jorge Motta

**Affiliations:** From the Gorgas Memorial Institute for Health Studies (JZ, VH, BG, JM); Centro de Biología Molecular y Celular de Enfermedades, Instituto de Investigaciones Científicas y Servicios de Alta Tecnología (MT); Región de Panamá Oeste, Caja de Seguro Social, Panama City, Panama (MT); and Institute of Specialized Analysis of the University of Panama, Miraflores, Panama City, Panama (WU).

## Abstract

In recent years, Panama has experienced a marked economic growth, and this, in turn, has been associated with rapid urban development and degradation of air quality. This study is the first evaluation done in Panama on the association between air pollution and mortality. Our objective was to assess the possible association between monthly levels of PM_10_, O_3_, and NO_2,_ and cardiovascular, respiratory, and diabetes mortality, as well as the seasonal variation of mortality in Panama City, Panama.

The study was conducted in Panama City, using air pollution data from January 2003 to December 2013. We utilized a Poisson regression model based on generalized linear models, to evaluate the association between PM_10_, NO_2_, and O_3_ exposure and mortality from diabetes, cardiovascular, and respiratory diseases. The sample size for PM_10_, NO_2_, and O_2_ was 132, 132, and 108 monthly averages, respectively.

We found that levels of PM_10_, O_3_, and NO_2_ were associated with increases in cardiovascular, respiratory, and diabetes mortality. For PM_10_ levels ≥ 40 μg/m^3^, we found an increase in cardiovascular mortality of 9.7% (CI 5.8–13.6%), and an increase of 12.6% (CI 0.2–24.2%) in respiratory mortality. For O_3_ levels ≥ 20 μg/m^3^ we found an increase of 32.4% (IC 14.6–52.9) in respiratory mortality, after a 2-month lag period following exposure in the 65 to <74 year-old age group. For NO_2_ levels ≥20 μg/m^3^ we found an increase in respiratory mortality of 11.2% (IC 1.9–21.3), after a 2-month lag period following exposure among those aged between 65 and <74 years.

There could be an association between the air pollution in Panama City and an increase in cardiovascular, respiratory, and diabetes mortality. This study confirms the urgent need to improve the measurement frequency of air pollutants in Panama.

## Abstract 2

Supplemental Digital Content is available in the text

## INTRODUCTION

Air pollution is an important public health problem that causes adverse health and economic consequences.^1–3^ In 2012, a report by the World Health Organization (WHO) determined that 3.7 million people died as a result of air pollution, especially in rapidly growing developing countries.^4^ Among the air pollutants, particulate matter with an aerodynamic diameter of <10 μm (PM_10_) is mostly associated with the air pollution in large cities. Nitrogen dioxide (NO_2_) and sulfur dioxide (SO_2_) are the primary air pollutants associated with vehicles.^5^ On the other hand, levels of ozone (O_3_) have been increasingly associated with climate change.^6^ According to the WHO, increased levels of these air pollutants lead to multiple adverse health effects.^7^

Epidemiology studies, primarily in Asia, have shown adverse health effects of short- and long-term exposure to air pollutants are associated not only with morbidity manifested by myocardial infarction or stroke, but also with mortality associated to diabetes, respiratory, and cardiovascular disease.^8–13^ In Latin America, published data related to the health effects of air pollutants are limited to Mexico, Brazil, and Chile.^14^ Based on these findings, many countries have identified air pollution as a public health problem because of the multiple evidence of its effect on population health and mortality.^3,15–17^ Some countries have gone further, implementing public measures to control air pollutants, with good results.^18–20^

According to the World Bank, Panama has had one of the highest rates of economic growth in Latin America in the last decade, which has been associated with rapid urban growth especially in Panama City.^21^ This rapid urban growth, in turn, has been linked to increased levels of air pollutants like particulate matter with an aerodynamic diameter of <2.5 μm (PM_2.5_), reaching on some days concentrations >25 μg/m^3^.^22^ Although levels of some air pollutants have been recorded for several years, there have been no published reports analyzing the association of air pollution with mortality in Panama or in Central America.

The purpose of this study is to assess the possible association between monthly levels of PM_10_, O_3_, and NO_2,_ and cardiovascular, respiratory, and diabetes mortality and also the seasonal variation of mortality in Panama City.

## METHODS

### Study Area

Panama City is Panama's largest city, with a total surface area of 280 km^2^, inhabited by ∼1.2 million people, which represent ∼40% of the total population of the country.^23^ Panama City is composed of the district of Panama and, partially, by the district of San Miguelito. Both districts account for 16 subdivisions (corregimientos).

### Procedures

We analyzed air pollution data of NO_2_ and PM_10_ from January 2003 to December 2013 and O_3_ data from January 2003 to December 2011 in Panama City by using a Poisson regression model. Data for O_3_ was not available for 2012 and 2013; therefore, these years were not included in the analyzed model. We could only use this time range for analysis because limited data on air pollutants were available before 2003. Also, the study only included Panama City because there were no air pollution data and lack of high-quality mortality data in other parts of the country. In this study, we also report a seasonality descriptive pattern by using a time series decomposition procedure.

The sample size consisted of a 132 monthly average measurements for NO_2_ and PM_10_ and 108 monthly measurements for O_3_. Two daily measurements, at 8AM and at 4PM, were averaged for a daily value. This process was performed twice a week. Then, all the measurements available in each month were averaged for a single monthly value. For NO_2_ and PM_10_, twice a week values were available. For O_3_, once or twice a week values were averaged (see *Weather and Air Pollution Data* section).

## DATA COLLECTION

### Mortality

Monthly mortality statistics from January 2003 to December 2013 were obtained from the National Institute of Statistics and Census. Panama City has the highest quality of mortality data in the country, were a qualified doctor certifies all deaths.^24^

The causes of death were coded according to the 10th International Classification of Diseases (ICD-10) and segregated into 3 categories: cardiovascular mortality (I00–I99), respiratory mortality (J00–J99), and diabetes mortality (E10–E14). We excluded Influenza (J10–J11) of respiratory mortality, as it was considered a potential confounding variable in the model. The Ministry of Health of Panama provided the data of influenza cases. Mortality was stratified according to 4 age groups: <65 years, 65 years to 74 years, 75 to 84 years, and ≥85 years.

### Weather and Air Pollution Data

For the study period, we obtained daily maximum and minimum temperatures taken between 12:00 h and 00:00 h in degrees Celsius (°C), percent relative humidity (%), precipitation in millimeters (mm^3^), wind speed at 10 m/s and calculated a monthly average for these weather variables. The Electric Transmission Company (ETESA) supplied all the weather data (without missing values). The temperature was obtained from the station of Hato Pintado #142–020, 45 meter elevation, 9°00′33N′′ latitude, 79°30′52W′′ longitude and the rest of weather data from the station of Tocumen #14402 WMO (World Meteorological Organization) # 78792, 14 meter elevation, 9°03′00′′N latitude, longitude 79°22′00′′W longitude.

**FIGURE 1:**
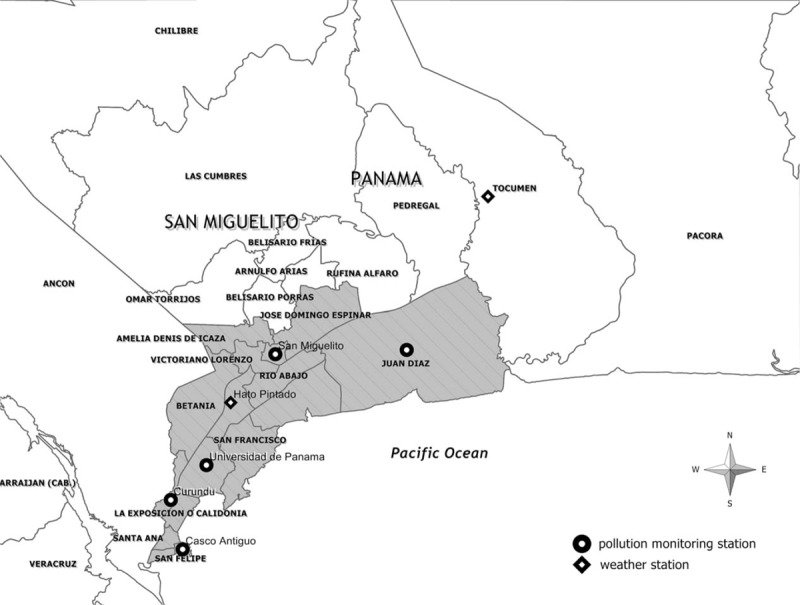
Map of Panama City showing the location of the weather stations and the 5 pollution-monitoring stations.

We utilized 5 air pollution-monitoring stations distributed in different representative locations of the city. For PM_10_ and NO_2,_ data were available twice a week (Monday and Thursday, or Tuesday and Friday) from the different air pollution-monitoring station. Forty values (8 per station by 5 stations) for PM_10_ and NO_2_ each month were averaged to produce a single monthly value, using a method similar to another study.^25^ The Institute of Specialized Analysis of the University of Panama, which provided the air pollution data, measured O_3_ once or twice per week; therefore, 20 to 40 daily measurements per month were average to obtain a representative single monthly value (see Figure 1).

The passive tubes technique was used for the measurement of O_3_ and NO_2,_ and a Harvard-type impactor was used for the measurement of PM_10._ All the stations used the same standards and measurement protocols.^26,27^ In 6 instances, there was a temporal interruption of a daily measurement; therefore we estimated the absent daily measurement by using the monthly mean of the contaminant of the pollutant-monitoring station where the value was not recorded.

## SEASONALITY

We calculated the seasonality of monthly mortality and pollutant levels for the period January 2003 to December 2013 by utilizing cardiovascular, respiratory, and diabetes mortality, and the levels of PM_10_, O_3_, and NO_2_. The seasonal component was calculated by the moving averages method, using data 6 months before and 6 month after the calculated month. We assumed that the pattern of variation remained constant from year to year (random error equal to zero) and that it could be quantified with index numbers (seasonal index) when the aggregation scheme was multiplicative. The seasonal index (SI) was used to summarize the behavior of the contaminants and disease variable per month.

### Single Pollutants Model

For the study period, a Poisson regression model, based on generalized linear models, was used to assess changes in mortality risk for cardiovascular, respiratory, and diabetes diseases and the association to the levels of O_3_, PM_10_, and NO_2_. The dependent variable was the monthly and total mortality stratified by the following age groups: <65 years, 65 to 74 years, 75 to 84 years, and ≥85 years. For the gender analysis we did not find an association between air pollution and mortality. The significance level was established at 0.05.

We established 2 dummy variables for the analysis of each pollutant (PM_10_≥40 μg/m^3^, O_3_ ≥20 μg/m^3^, NO_2_ ≥20 μg/m^3^) to determine the point considered to be the highest level of contamination.^28^

We used a cut-off value of 20 μg/m^3^ for O_3_ as suggested by a meta-analysis result.^29^ For PM_10_ (≥40 μg/m^3^) and NO_2_ (≥20 μg/m^3^) we used the cutoff values using a methodology similar to other reports.^15,30^ Briefly, for PM_10_ and NO_2_, we looked for the air contaminant value in which we found an effect with an adequate sample size. These values were higher than others used previously.^5,12,31^ Lower cutoff values could not be used as lower values of PM_10_ and NO_2_ were scarce to perform the analysis. The validity of each model was evaluated by the omnibus test for logistic regression and by the likelihood ratio test. Each model was controlled for relative humidity, precipitation, 10-meter wind speed, mean temperature, linear trend in mortality, and influenza cases (see equation in supplementary data). We used the influenza variable as a reference point (odds ratio: 1.00). The effect of environmental pollutants on mortality was also evaluated using 1- and 2-month mortality lags. The results are presented as a percentage change in mortality with their respective 95% confidence intervals (CI).

Spearman's rank correlation coefficient analysis was performed using monthly values of PM_10_ O_3_, NO_2_, and relative humidity, average temperature, and wind speed, in order to assess the degree of correlation between these variables. Data were analyzed using SPSS 19.0 Statistical Package (IBM, Armonk, NY) and plotted in GraphPad Prism 6 (La Joya, CA). An ethics committee reviewed and approved the study.

## RESULTS

### Mortality and Air Pollution Data

From 2003 to 2013, there were 18,468 cardiovascular, 5709 respiratory, and 4404 deaths due to diabetes in the 16 subdivisions (corregimientos) of Panama City. Table 1 summarizes the mean monthly mortality, air pollution levels, average temperature, relative humidity, and wind speed in the city. The mean monthly mortality attributed to cardiovascular and respiratory diseases and diabetes was 139.9 deaths/month, 43.2 deaths/month, and 33.4 deaths/month, respectively. The mean monthly concentrations for PM_10_, O_3_, and NO_2_ were 13.8 μg/m^3^, 43.8 μg/m^3^, and 28.9 μg/m^3^ with interquartile ranges (IQR) of 5.97 μg/m^3^, 11.28 μg/m^3^, and 5.82 μg/m^3^, respectively. The mean temperature was 27.9°C with an IQR of 1.12°C. The average monthly relative humidity was 74.9% with an IQR of 11 and the mean 10-m wind speed was 1.82 m/s with an IQR of 0.64 m/s.

**TABLE 1:**
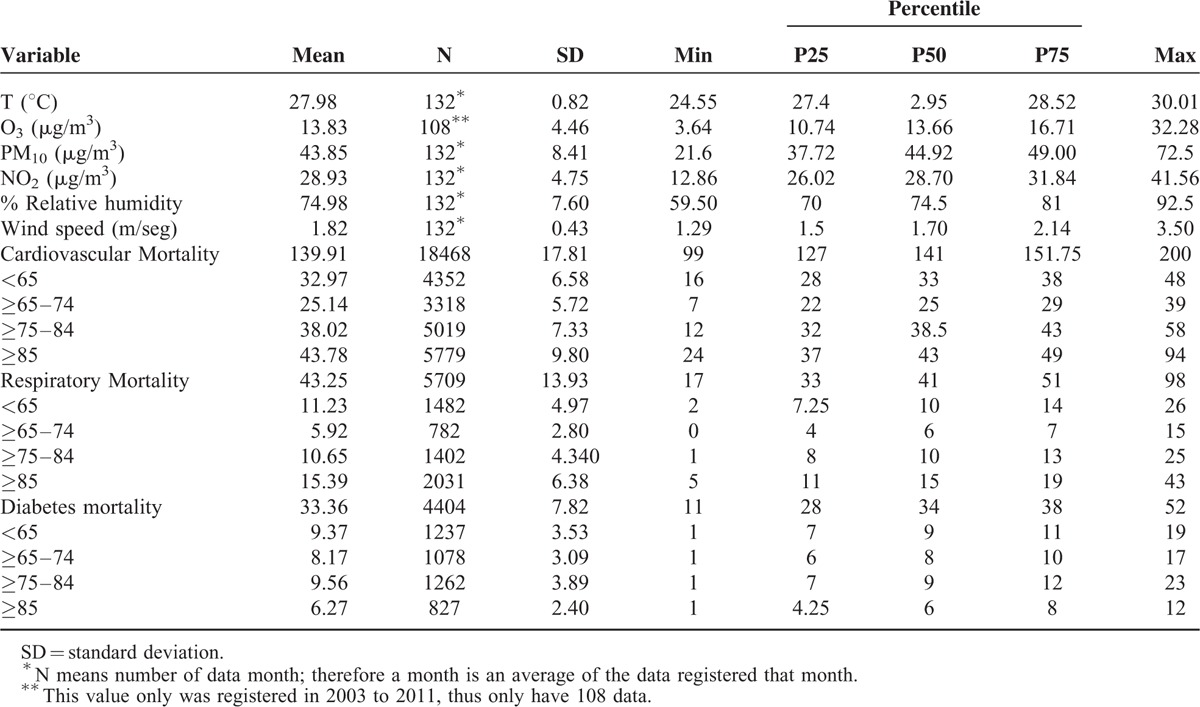
Descriptive Summary for the Study Period, Monthly Deaths, Air Pollutants, and Weather Conditions in Panama City from 2003 to 2013

### Seasonality

**FIGURE 2:**
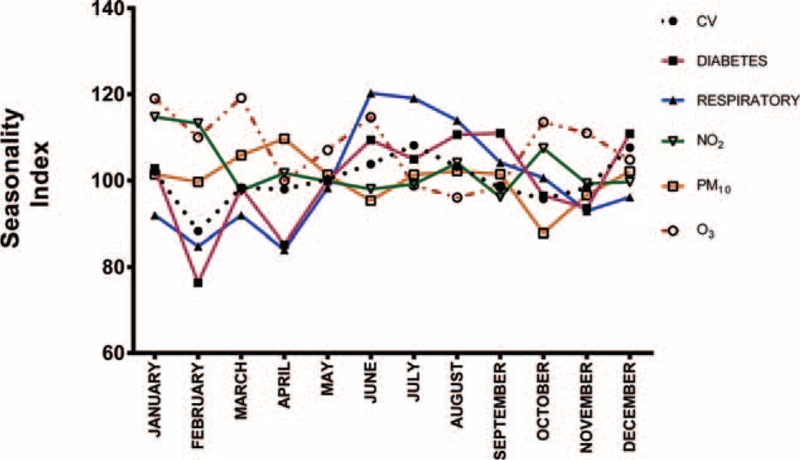
Monthly mortality and air pollutants seasonality in Panama City from 2003 to 2013. CV = cardiovascular mortality, NO_2_ = nitrogen dioxide, O_3_ = ozone, this value only was registered in 2003 to 2011, PM10 = particulate matter ≤10 μg.

Figure 2 summarizes mortality and pollution SI. The lowest SI of cardiovascular disease (SI 88) and diabetes mortality (SI 76) occurred during February. The peak of mortality for cardiovascular disease occurred in June (SI 108) and for diabetes in September (SI 110). As for respiratory mortality, the lowest point occurred in April (SI 83) the highest value occurred in June (SI 120). O_3_ pollution presented its highest SI in March (SI 119) and its lowest SI in August (SI 96), whereas PM_10_ registered its highest SI in April (SI 109) and its lowest SI in October (SI 88). NO_2_ reached its highest SI level in February (SI 113), but there was less variation in NO_2_ levels during the year.

### Results of the Model

**FIGURE 3:**
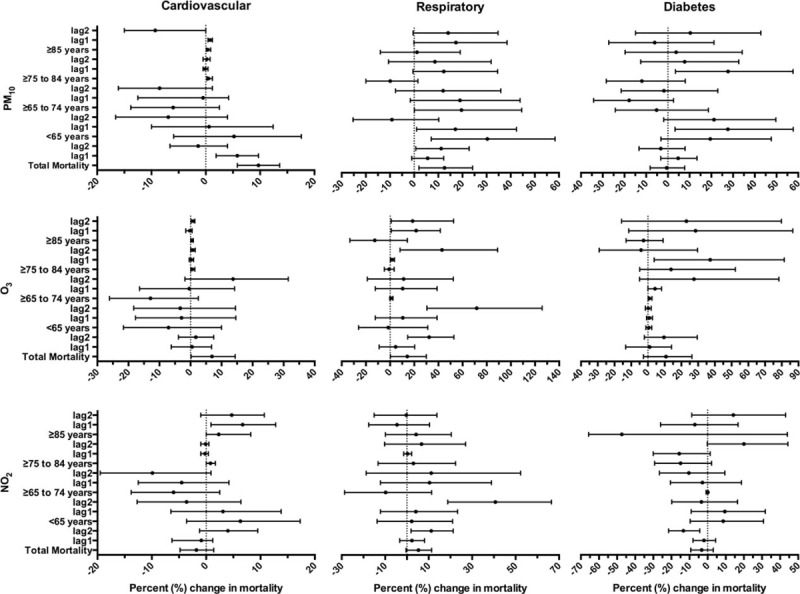
Percentage change (mean and 95%CI) in mortality associated with an increase in ≥40 μg/m^3^ PM_10_ (monthly lag 0, 1, 2), ≥20 μg/m^3^ in O_3_ (monthly lag 0, 1, 2), and 20 μg/m^3^ in NO_2_ (monthly lag 0, 1, 2) in Panama City from 2003 to 2013. ∗The association with ozone only was analyzed from 2003 to 2011. Supplemental Digital Content. Odds ratio values (mean and 95%CI) in mortality associated with an increase in ≥40 μg/m^3^ PM_10_ (lag 0, 1, 2), ≥20 μg/m^3^ in O_3_ (lag 0, 1, 2), and 20 μg/m^3^ in NO_2_ (lag 0, 1, 2) in Panama City from 2003 to 2013.CI = confidence interval, NO_2_ = nitrogen dioxide, PM_10_ = particulate matter ≤10 μm.

Figure 3 shows the subacute association between total mortality and air pollution (PM_10_, O_3_, and NO_2_) from 2003 to 2013 in Panama City, stratified by age and by 1 to 2 month interval lag periods (see Table 1 in supplementary file). For PM_10_ ≥ 40 μg/m^3^, the study revealed an increase of 9.7% (CI 5.8% to 13.6%) in total cardiovascular mortality and of 5.8% (CI 1.9–9.7%) 1 month after the time of exposure and we did not obtain a statistically significant association 2 months after the exposure. Regarding respiratory mortality, there was an increase of 12.6% (CI 0.2–24.2%) in total mortality, a 5.6% (CI 0–12.3%) increase after a 1-month lag period, and an 11.2% (CI 0.7–22.8%) increase after a 2-month lag period from the time exposure. When stratified by age, respiratory mortality increased to 30% (CI 7.1–58.0%) in the <65 year-old age group. For total diabetes mortality and after 1- and 2-month lag periods we observed no statistically significant association. When stratified by age, the <65 year-old group showed a 27.6% (CI 3.3–57.6%) increase in mortality after a 1-month lag period from the time of exposure (see table with odd ratios in supplemental content).

O_3_ levels ≥20 μg/m^3^ were associated with an increase of 6.9% (CI 0.2–14.4%) without lag period, a 0.5% (CI −0.062–6.8%) increase after 1-month lag period and a 1.7% (CI −0.039% to 7.5%) increase in cardiovascular total mortality after a 2-month lag period from the time exposure. For total respiratory mortality, there was a 14.2% (CI 0.3–30%) increase without lag period, a 4.6% (CI –0.08% to 20%) increase after a 1-month lag period and a 32.4% increase (CI 14.6–52.9%) after a 2-month lag period after the time of exposure. When stratified by age, there was a greater risk of respiratory mortality in the <65 year-old age group. For total diabetes mortality and after a 1- and 2-month lag periods we did not obtain a statistically significant association. However, when stratified by age, the ≥65 to 74 years group showed a 1% (CI 0.1–2.4%) increase in mortality after a 1-month lag period.

Levels of NO_2_ ≥20 μg/m^3^ were associated with an increase in cardiovascular mortality of 2.3% (CI 0.1–8.2%) in the ≥85 year-old group and a 6.7% (CI 0.9–12.8%) increase after a 1-month lag period. For total respiratory mortality, we did not obtain a statistically significant association without lag period and after a 1-month lag period, although we observed an increase of 11.2% (CI 1.9–21.3%) after a 2-month lag period. In the ≤65 age group we observed a 40.7% (CI 18.8–66.5%) increase after a 2-month lag period from the time of exposure. NO_2_ was not significantly associated with an effect on total diabetes mortality, after and without lag periods.

Table 2 shows a Spearman correlation analysis between environmental pollutants and weather variables. We observed that environmental pollutants had an inverse, but not statistically significant (*P*>0.05), correlation with wind speed. The levels of O_3_ and PM_10_ were inversely correlated with relative humidity levels. The levels of PM_10_ were inversely correlated with relative humidity (*r* = 0.363, *P* = 0.03). O_3_ and NO_2_ were also inversely correlated (*r* = −0.267, *P* = 0.005).

**TABLE 2:**
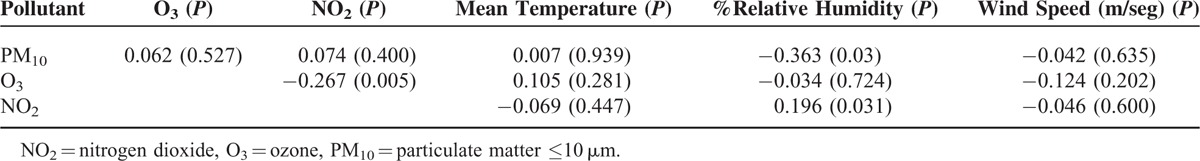
Spearman Correlation Coefficients Between Air Pollutants and Weather Variables. The *P* Values Are Represented in Parentheses After Each Correlation Coefficient

## DISCUSSION

This is the first study done in Panama and Central America that has made an assessment of the possible association between air pollutants and a subacute increase in cardiovascular, respiratory, and diabetes mortality. Using statistical models that assess the subacute individual impact of each pollutant, we conclude that PM_10_, O_3_, and NO_2_ represents another factor together with comorbidities, treatment adherence, age, and socioeconomic status that could be increasing mortality statistics in Panama City, Panama.

The pathophysiologic effects of air pollutants have been evaluated in several controlled animal studies and, because of ethical considerations, in a few human studies. Studies have shown that inhalation of particulate pollutants cause an inflammatory response of the airways and of the endothelium, associated to neutrophil and monocytes migration, a rise in the levels of inflammatory cytokines such as TNF-α, IL-1, and IL-6, and an increase in oxidative stress.^32–36^ Rats exposed to concentrated ambient pollutants have shown a predisposition to longer inflammatory effects of their respiratory airways.^37^ In humans, hemodynamic studies have shown significant acute blood pressure increases after exposure to air pollutant particles or to controlled levels of O_3._^38–40^

In patients with diabetes due to own endothelial alteration of the disease, the inflammatory response product of air pollution and cardiovascular effect is greater, predisposing to the development of cardiovascular events and inflammation of the respiratory epithelium, which seems to be more evident in patients <65 without adequate control.^41–43^ Other studies have shown an increased risk of developing a prothrombotic state after exposure to high levels of airborne particulate contaminants, which is more marked in diabetics.^42,44–46^

### PM_10_

As described in this study, many investigations have demonstrated an association between the short- and long-term exposure to high levels of PM_10_ and an increased risk of cardiovascular and respiratory mortality.^12,15,30,47,48^ A study done in 2 Austrian cities, utilizing a similar statistical analysis as in this study, showed an increase mortality risk from cardiovascular disease of 2.0% (95% CI, 0.9–3.1%) and an increase mortality risk from respiratory disease of 3.0% (95% CI, 0.5–5.5%) after a 15-days of exposure to PM_10_.

The effect of the air pollutants is more evident in respiratory and cardiovascular mortality without lag period and after 1 month lag period. The ≤65 years and ≥85 year-old groups appear to be more vulnerable. This may be due to a combination effect of air pollution and other factors including socioeconomic status, comorbidities, lifestyle, and treatment adherence. We did not observe a statistically significant association when exploring a 2-month lag period.

When compared to other studies, the marked increase risk in respiratory mortality due to PM_10_ noted in our study (12.6%) could be explained by the exposure to high levels of PM_10_ (≥40 μg/m^3^) registered not frequently in the city and the increased exposure time.^49,50^

In our study, as well as in another report, the association of PM_10_ to respiratory mortality was more pronounced in ≤65 years and ≥85 year-old groups, which because of age-related changes, have a higher prevalence of respiratory pathologies that might be worsened by exposure to this pollutant.^50^

For diabetes mortality we did not find a consistent association with air pollutants. Some experimental studies suggest increases in prothrombotic states that could be associated with mortality events, but more epidemiological studies are needed to conclude the subacute effect of PM_10_ in diabetes mortality.^42,51^

### O_3_

Contrary to other studies that did not find an association between exposure to O_3_ and mortality, our study found a significant association between levels of O_3_ ≥20 μg/m^3^ and mortality from total cardiovascular mortality (6.9%) and total respiratory mortality (14.2%) disease.^49^ The association of O_3_ exposure to respiratory mortality was observed after 1- and 2-month lag periods in the ≥75 to 84 age group. This relation could suggest that the subacute exposure to O_3_ has a possible effect, which is more evident in older age groups, a study that evaluate the acute exposure of O_3_ in older age groups find an increase in cardiovascular and respiratory mortality.^52^ This finding is in line with the acute and subacute injury seen in experimental studies that have evaluated the effects of O_3_ exposure on the respiratory and cardiovascular systems of rats and in human tissue culture studies.^51,53^

### NO_2_

Research studies assessing the short- and long-term effects of NO_2_ on human health have shown an increased risk of cardiovascular, respiratory, and diabetes mortality for NO_2_ levels ≥10 μg/m^3^.^5,15,31,54^ One study also demonstrated, after subacute exposure of NO_2_, an increase in mortality of 4.6% (95% CI, 01.6–4.1%) from cardiovascular disease and an increase in mortality of 6.7% (95% CI, 2.7–10.8%) from respiratory mortality.^49^

Our study as have the above-mentioned studies, found an association between increased levels of NO_2_ and cardiovascular mortality but only in the ≥74 year old group, as well as an increase in respiratory mortality only in the ≥65 to 74-year-old group. Furthermore, in both groups, the increase in mortality was shown after exposure to NO_2_ after a 2-month lag period. We believe the difference found in our study compared to others, concerning the health effects of NO_2_, might be due to the sampling frequency of NO_2_ in our study. A more accurate assessment of the relation of NO_2_ exposure to mortality could have been achieved with daily measurements.

Seasonal mortality patterns have been studied in several countries through various analytical models. Some studies have established an association of seasonal mortality with weather variables such as temperature and wind speed whereas others have demonstrated the role of wind speed, wind direction, and other weather variables on the levels of air pollutants.^17,55^ Our study revealed a seasonal mortality pattern characterized by a lower mortality SI for cardiovascular disease and diabetes during January to March. We hypothesized that the northeast trade winds, that are more prominently present during the months of January and February (early dry season of Panama), could have a cleansing effect of air pollutants of the city and that this wind effect could have played a role in the observed reduction of mortality during those months. Although our analysis showed a slight inverse relation between the levels of air pollutants and wind speed, this inverse relation did not reach statistical significance. NO_2_ and O_3_ that are intimately linked through atmospheric chemistry and continuous interchange over very short timescales.^56^ In our study we observed a slightly negative correlation (*P* < 0.05). This correlation should be addressed in other studies.

Our study was limited by the sampling frequency of air pollution data and by the availability only of monthly mortality data, which was used to analyze the possible association between air pollution and mortality in Panama City. Daily sampling of pollutants and daily mortality data surely would have given us a more precise assessment of the relation between mortality from cardiovascular disease, respiratory disease, and diabetes and air pollution. The other limitation was the frequency of weekly sampling and the year period of measurements of O_3_ levels, which extended from 2003 to 2011, whereas the period of measurement of the other contaminants was from 2003 to 2013. This reduced the sample size in the analysis might have decreased the capacity to determine more precisely the deleterious effect of O_3_ shown in the study. Also, the irregular time of observation of O_3_ and the other 2 pollutants could have affected the correlation analysis between environmental pollutants and weather variables.

Panama is a country that has sustained high economic growth and a rapid urban expansion in recent years, and it needs better monitoring of air pollutant levels, its health impacts, and the control of its sources. This study showed a possible deleterious association between the air pollution in Panama City and cardiovascular, respiratory, and diabetes mortality. Also, it confirms the urgent need to improve the sampling frequency of air pollutants in the country. Finally, we believe that this will allow us to precisely address the health effects, with the aim of developing policies to protect the citizens.

## Supplementary Material

Supplemental Digital Content
