# Supplementary material for: Assessment of the Possible Association of Air Pollutants PM10, O3, NO2 With an Increase in Cardiovascular, Respiratory, and Diabetes Mortality in Panama City: A 2003 to 2013 Data Analysis
Source: Medicine (Baltimore). 2016 Jan 15;95(2):e2464. doi: 10.1097/MD.0000000000002464 (PMC4718270; doi:10.1097/MD.0000000000002464)
Supplement: Supplemental Digital Content [file medi-95-e2464-s001.doc]

**Table 1.** **Odds ratio values (mean and 95% CI) in mortality associated with an increase in ≥40 µg/m^3^ PM_10_ (lag 0,1,2), ≥20µg/m^3^ in O_3_ (lag 0,1,2) and 20µg/m^3^ in NO_2_ (lag 0,1,2) in Panama City from 2003-2013.**

|  | **PM10** | | | **Ozono** | | | ***NO2*** | | |
| --- | --- | --- | --- | --- | --- | --- | --- | --- | --- |
| **Mortallity** | **Lag 0** | **Lag 1** | **Lag 2** | **Lag0** | **Lag1** | **Lag2** | ***Lag0*** | ***Lag1*** | ***Lag2*** |
| **Cardiovascular (I00-I99)** |  |  |  |  |  |  |  |  |  |
| Total | 1.097(1.058,1.136) | 1.058(1.019,1.097) | 0.986(0.934,1.040) | 1.069(1.001,1.144) | 1.005(0.938,1.068) | 1.017(0.961,1.075) | 0.982(0.952,1.014) | 0.991(0.937,1.012) | 1.040(0.988,1.095) |
| <65 years | 1.052(0.941,1.176) | 1.006(0.900,1.124) | 0.931(0.834,1,040) | 0.929(0.784,1.100) | 0.971(0823,1.146) | 0.967(0.817,1.145) | 1.063(0.964,1.173) | 1.031(0.935-1.138) | 0.964(0.873-1.064) |
| ≥65-74 | 0.940(0.862-1.025) | 0.995(0.875-1.042) | 0.915(0.839-1.012) | 0.871(0.739-1.025) | 0.996(0.836,1.142) | 1.137(0.982,1.315) | 0.940(0.862,1.025) | 0.955(0.875,1.042) | 0.901(0.805,1.009) |
| ≥75-84 | 1.005(1.002,1.012) | 0.999(0.995-1.004) | 1.002(0.995-1.008) | 1.006(1.001-1.014) | 1.003(0.996-1.010) | 1.008(1.001-1.015) | 1.008(1.000,1.017) | 0.998(0.990,1.004) | 0.999(0.990,1.005) |
| ≥85 | 1.004(1.001,1.009) | 1.008(1.004,1.012) | 0.907(0.850,1.000) | 1.005(1.001,1.009) | 0.998(0.985,1.005) | 1.009(1.002,1.014) | 1.023(1.000,1.082) | 1.067(1.009,1.128) | 1.047(0.990,1.107) |
| **Respiratory (J00-J99)** |  |  |  |  |  |  |  |  |  |
| Total | 1.126(1.02,1.242) | 1.056(0.99,1.123) | 1.112(1.007,1.228) | 1.142(1.003,1.300) | 1.046(0.91,1.203) | 1.324(1.146,1.529) | 1.053(0.996,1.114) | 1.023(0.967,1.081) | 1.112(1.019,1.213) |
| <65years | 1.303(1.071,1.584) | 1.171(1.004,1,424) | 0.908(0.747,1.102) | 0.985(0.739,1.312) | 1.105(0.878,1.389) | 1.717(1.305,2.259) | 1.022(0,863,1.210) | 1.041(0.878,1.233) | 1.407(1.188,1.665) |
| ≥65-74 | 1.196(1.001,1.446) | 1.190(0.984,1.439) | 1.120(0.923,1.359) | 1.006(0.998,1.024) | 1.104(0.878,1.389) | 1.112(0.812,1.523) | 0.902(0.713-1.114) | 1.104(0.878,1.389) | 1.112(0.812,1.523) |
| ≥75-84 | 0.901(0.800,1.016) | 1.123(0.995,1.346) | 1.086(0.894,1.319) | 0.992(0.952,1.034) | 1.019(1.003,1.036) | 1.430(1.082,1.890) | 1.030(0.867,1.224) | 1.003(0.985,1.022) | 1.067(0.897,1.270) |
| ≥85 | 1.012(0.860,1.191) | 1.173(0.999,1.385) | 1.141(0.996,1.348) | 0.874(0.668,1.143) | 1.216(1.000,1.416) | 1.187(1.005,1.526) | 1.042(0.901,1.204) | 0.954(0.824,1.104) | 0.819(0.719,1.138) |
| **Diabetes (E10-E14)** |  |  |  |  |  |  |  |  |  |
| Total | 0.994(0.918,1.077) | 1.047(0.967,1.134) | 0.967(0.867,1.079) | 1.108(0.973,1.263) | 1.01(0.867,1.141) | 1.096(0.978,1.295) | 0.967(0.907,1.031) | 0.98(0.919,1.044) | 0-866(0.784,0.957) |
| <65 years | 1.195(0.968,1.476) | 1.276(1.033,1.577) | 1.212(0.981,1.497) | 1.005(0.988,1.023) | 1.009(0.992,1.027) | 1.002(0.985,1.020) | 1.086(0.903,1.308) | 1.095(0.909,1.318) | 0.966(0.802,1.165) |
| ≥65-74 | 0.948(0.758,1.186) | 0.822(0.658,1.026) | 0.982(0.785,1.230) | 1.009(1.001,1.024) | 1.042(1.000,1.042) | 1.275(0.95,1.783) | 1.122(0.082,1.215) | 0.971(0.794,1.187) | 0.896(0.734,1.095) |
| ≥75-84 | 0.880(0.716,1.080) | 1.277(1.035,1.576) | 1.077(0.875,1.326) | 1.138(0.951,1.523) | 1.372(1.037,1.815) | 0.959(0.709,1.297) | 0.850(0.707,1.022) | 0.843(0.700,1.014) | 1.200(0.998,1.443) |
| ≥85 | 1.038(0.803,1.342) | 0.939(0.728,1.212) | 1.103(0.851,1.428) | 0.974(0.869,1.091) | 1.285(0.885,1.866) | 1.230(0.842,1,798) | 0.526(0.343,1.440) | 0.929(0.740,1,168) | 1.142(0.912,1.431) |
